# Agent-based modeling to estimate the impact of lockdown scenarios and events on a pandemic exemplified on SARS-CoV-2

**DOI:** 10.1038/s41598-024-63795-1

**Published:** 2024-06-11

**Authors:** Christian Nitzsche, Stefan Simm

**Affiliations:** 1 University Medicine Greifswald, Institute of Bioinformatics, Greifswald, 17489 Germany; 2 Coburg University of Applied Sciences, Institute of Bioanalysis, Coburg, Germany

**Keywords:** Agent-based modeling, SARS-CoV-2, COVID-19, Mitigation measure simulation, Individual transfection risk, Lockdown scenarios, Computational models, Risk factors, Epidemiology

## Abstract

In actual pandemic situations like COVID-19, it is important to understand the influence of single mitigation measures as well as combinations to create most dynamic impact for lockdown scenarios. Therefore we created an agent-based model (ABM) to simulate the spread of SARS-CoV-2 in an abstract city model with several types of places and agents. In comparison to infection numbers in Germany our ABM could be shown to behave similarly during the first wave. In our model, we implemented the possibility to test the effectiveness of mitigation measures and lockdown scenarios on the course of the pandemic. In this context, we focused on parameters of local events as possible mitigation measures and ran simulations, including varying size, duration, frequency and the proportion of events. The majority of changes to single event parameters, with the exception of frequency, showed only a small influence on the overall course of the pandemic. By applying different lockdown scenarios in our simulations, we could observe drastic changes in the number of infections per day. Depending on the lockdown strategy, we even observed a delayed peak in infection numbers of the second wave. As an advantage of the developed ABM, it is possible to analyze the individual risk of single agents during the pandemic. In contrast to standard or adjusted ODEs, we observed a 21% (with masks) / 48% (without masks) increased risk for single reappearing participants on local events, with a linearly increasing risk based on the length of the events.

## Introduction

The SARS-CoV-2 pandemic has led to the implementation of a range of mitigation measures, including keeping distance, wearing masks and closure of public places and events. However, there is considerable uncertainty surrounding the effectiveness of these measures and combination of measures. Modeling of infectious diseases has been shown during COVID-19 to play a pivotal role in predicting the course of the pandemic^1^. The investigation of pandemic courses can be approached by either the use of equation-based models, such as ordinary differential equations (ODE) and partial differential equations (PDE), or equation-free models, such as agent-based models (ABM). In general, these modeling approaches have their own strengths and weaknesses, allowing the investigation of different aspects, especially in the spread of an infectious disease^2–4^. For such predictions ABMs are able to explain the effects of interactions between single humans (agents) by simulating them individually. The sum of all these micro-level interactions is what leads to observable macro-level effects. By this, agent groups and their distribution permit the approximation of more realistic processes due to individualized simulation, as seen in other life science domains, simulating enzyme activations in cells^5^. The simulation of single interactions between two agents enables ABMs to have a heterogeneous agents environment, including distributions of agent groups at different places and their individual interactions at these places specifically. However, this detailed view results in ABMs having a long run time that increases with the population size. Conversely, equation-based models based on ODEs, such as the SIR model by Kermack and McKendrick^6^ simplify interactions on a population basis and make assumptions on a large-scale level, leading to a model that is fully described by a set of equations. In particular, in the case of ODEs, this implicitly leads to the assumption, that the simulation domain is homogeneous. Consequently, these models are less computationally intensive and the run time is usually independent of the population size. Since 1760, when smallpox was studied by Bernoulli, the interest in predicting and understanding the spread of infectious diseases has been increased. This has led to the creation or adaptation of new methods and models based on new data sources and amounts of data^7^.

So far, for modeling the spread of infectious diseases ABM and ODE approaches have been used to shed light on different aspects. For instance, ODE approaches were utilized during the 2014 Ebola outbreak in western Africa to ascertain optimal control theories^8,9^ and the dynamic interactions between susceptible and infected populations^10^. In ODE models usually SIR, SEIR or SEID have been used including principle features about deterministic contact tracing numbers. These models can be utilized for simulations by modifying the parameter regimes. One hallenge with numerous ODE models in relation to the Ebola outbreak was that some of the assumptions lacked clear justifications or were not aligned with the disease's characteristics^11^. For this reason, ABMs have been developed as well for the Ebola disease with a different focus as it was possible to use contact networks^12^ for more differentiated spreading simulations in small-world network models. This allowed the simulation of the evolution of the epidemic with dynamic adaptations due to equation-free approaches. In another ABM, the movement of individuals was taken into consideration to understand the transmission of the disease in different environments and events like funerals^13^. For the modeling of the spread of SARS-CoV-2 both ODE models and ABMs have been successfully applied to predict the pandemic course and are important for political decisions about adjustments to a pandemic or the proposal of control strategies to prevent an increased infection^14–16^. Equation-based models such as the SIR model and related compartmental models were more often used for country-wide simulations, assuming a well-mixed homogeneous population^17^. Extensions of these compartmental models, such as reaction-diffusion-systems^18,19^ as an example for PDEs, are also able to take population densities into account with a country-wide spatial resolution. There are also approaches to combine microscopic (ABM) and macroscopic (ODE) models in order to combine the advantages of both^1^.

Recent reviews about forecasting models for COVID-19 summarized models based on strengths and weaknesses without mentioning ABM^20^. Nevertheless, there are also articles describing potential issues, such as the heterogenous shapes of epidemic dynamics^21^, particularly in the case of the COVID-19 pandemic. As a potential solution ABMs were developed focusing on a limited detailed view on different intervention measures (COVASIM^22^) or on a specific region^23^. These models often incorporate data from Google Maps or Open Street Map^24^. Further, such models were even able to evaluate the economic effects of different combinations of mitigation measures^25,26^. This allowed on a smaller scale the investigation of local transmissions of the virus in specific indoor settings during the lockdown by 2D grids^27^ or on a neighbourhood-scale^28^. The modeling kits COVASIM^22^ , COMOKIT^29^ and Epiabm^30^ give many options in analyzing mitigation policies against COVID-19, with a particular focus on this pandemic. Unlike COMOKIT, our ABM is simplified and written in Python like COVASIM, to make it easily adjustable for future zoonotic pandemic situations. In contrast to COVASIM, our model does not consider pharmaceutical mitigation measures. In contrast to several ABM models published in 2024^30–34^, our model is not based on a specific region or country (USA^31,34^, China^32^). Instead, it has been developed for abstract small city size communities, allowing for easy adjustments without much prior knowledge about parameters. This is in contrast to ABMs, where the parameters are often common knowledge. The current ABM models from Florida utilise synthetic maps to simulate the location of Ocala^31^ or base their ABM assumptions on resource-dependent communities like Alaska, focusing on factories^34^. Other ABMs focus on the spread of COVID-19, influenced by human mobility^32^ or expand their model to small-world networks^33^. The objective of our model is to be as abstract as possible at the scale of small cities, focusing on the opening or closing of specific places such as events. With the exception of the Mob-Cov^32^ and our model, which is publicly available on GitHub (https://github.com/Stegobully/Covid19_ABM), the other models are also written in Python but are not easily publicly available. Our model focuses on events by adding parameters such as frequency or duration. Additionally, it offers the ability to test lockdown scenarios, such as closing specific primary care facilities and adjusting the length of stay (for example in supermarkets) to make the effects of lockdown scenarios more transparent for citizens by calculating the individual infection risk. Conversely, the model does not consider pharmaceutical interventions or vaccinations, nor can it predict longer pandemics, including virus evolution or effects on future waves.

Currently missing in relation to the COVID-19 pandemic is a good forecast and prediction of lockdown scenario effects in different regions and using different mitigation measures. By this, it would be possible to simulate the particular efficiency of single mitigation measures like closing special places and even more fine grained parameters like amount of people at a time at the place and the influence to this mitigation. For such approaches ABMs can be advantageous to describe the real world behavior of humans^35^ of specific areas more accurately and dynamically based on individual components^4^. Another aspect is the handling of stochasticity in the system. In ABMs the effects of stochasticity are applied directly to agents, which might be important especially for discrete decisions (such as moving or staying at a place) instead of adding a noise term to the equation-based model^35^. At least to our knowledge, the implementation of a more abstract city ABM not restricted to a specific city or area^24^ is still missing in the context of COVID-19. For this, we implemented an ABM which represents an abstract city of a specific size and relatively simple agent groups to investigate the influence of different lockdown scenarios and further focusing on the influence of events and their related parameters on the individual risk during the COVID-19 pandemic. As a control for our predictions, we compared our ABM specialized to German area distribution of shops and agent roles with the lockdown scenarios and infections of Germany as well as with a standard ODE model for comparison. For the use case of our ABM we analyzed the effects of three different lockdown scenarios. These include combinations of mitigation measures like social distancing (including keeping distance and wearing masks) and closing of specific places as there were lots of discussions in the past. Further, our ABM even allowed to simulate the influence of local events and the effect of parameters like size, duration, frequency and reappearing attendees on the course of the pandemic. In the end, this allowed to get correlations between the length of events and the single individual risk to get infected as reappearing attendees, resulting in a risk increase of 21% for participants of events where everybody is wearing a mask and keeping distance and 48% for participants of events where nobody is wearing a mask or keeping distance.

## Materials and methods

### Infection model for COVID-19

**Figure 1:**
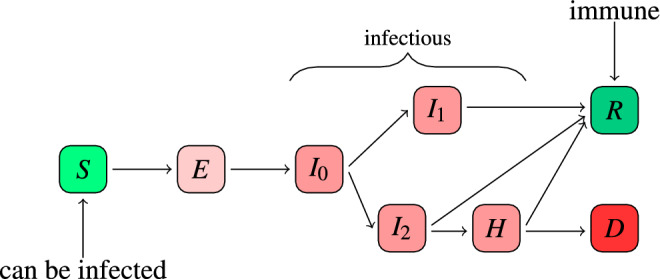
Flowchart for the possible states of an agent. Only agents in one of the states $\{ I_0, I_1, I_2, H\}$ are able to infect susceptible agents (state *S*) if they have contact with each other.

An adapted version of a classic SEIR approach (Fig. 1) including eight states was implemented to model the course of COVID-19. Similar adaptations were already published in different articles^36–38^. Since there is no natural immunity known against SARS-CoV-2, at the beginning of a pandemic, the majority of the agents will be susceptible to the virus (state *S*) and only a few agents have already been exposed to the virus (state *E*). Even before showing symptoms, agents can be infectious, which is represented by the state $I_0$. From state $I_0$ agents can change to $I_1$ if agents stay asymptomatic or to state $I_2$, if they are showing symptoms. In both cases they remain infectious. Agents in the asymptomatic state $I_1$ will recover for sure and change their state to *R* with time. Agents in state $I_2$ can either recover (state *R*) or get hospitalized (state *H*). Hospitalized agents can either recover (state *R*) or die (state *D*). Once recovered, agents in our model stay immune for the remaining simulation.

The length of stay in each state is modeled by using random numbers from a logistic distribution. We set the mean incubation time to 4 days (states $E \rightarrow I_0$) and it is expected that patients in state $I_0$ can be infectious 2 days before showing symptoms and changing to $I_1$ or $I_2$. Furthermore, it is stated by the Robert Koch Institute^39^, that patients with symptoms are on average infectious for 7 days (states $I_2 \rightarrow R$ or *D*) and we take the same assumption for asymptomatic cases (states $I_1 \rightarrow R$)). The average time in hospitals apart from ICU for COVID-19 patients is stated to be 7 to 10 days and in ICU with a maximum of needed care people stay in average 18 days (according to the Robert Koch Institute^39^). Given these numbers and because we do not differentiate the severity for hospitalized patients, we assume an average hospitalization time of 10 days (states $H \rightarrow R$ or *D*) in our model.

If there are two branches for choosing the new state, the new state is chosen according to given fractions (see Table 1). For the transition $I_0 \rightarrow I_1$ or $I_2$ we set the proportion of asymptomatic cases to $40 \%$. This is in line with the Robert Koch Institute^39^ ($15\% - 45\%$) and Oran and co-workers^40^ ($40\% - 45\%$). Further we estimated from infection numbers in the early phase of the pandemic in March and April 2020, that $\sim 17 \%$ of the symptomatic cases get hospitalized (transition from $I_2 \rightarrow H$).

**Table 1:** Disease parameters.

Parameter	Value
Mean time in state *E*	4 days
Mean time in state $I_0$	2 days
Mean time in state $I_1$	7 days
Mean time in state $I_2$	7 days
Mean time in state *H*	10 days
Fraction asymptomatic	40 %
Fraction hospitalized	17 %
IFR in hospitals	4.9 %

Describes the used parameter (column 1) and their default value (column 2) for the states from Fig. 1 (upper: time; lower: fraction probability).

Given the fractions for asymptomatic cases and hospitalized cases and assuming, that the overall infection fatality rate (IFR, death rate for infected agents) for COVID-19 is about $0.5\%$ (between $0.35\%$^41^ and $0.5\% - 1\%$^42^), we can obtain an estimate for the IFR of hospitalized agents in our model. Since in our model the only way to die is by being hospitalized and $17 \%$ of the symptomatic cases are getting hospitalized, we can use this to estimate that $4.9\%$ of the hospitalized agents die (state $H \rightarrow D$). Besides the states of an agent, we distinguish between normal-spreaders and multi-spreaders. According to the literature we assume that $19\%$ of the infectious agents are responsible for $80\%$ of all new infections^43^ and initialize our model with a proportion of $19\%$ multi-spreaders and $81\%$ normal-spreaders, regardless of the agent type. Multi-spreaders are included by giving them more contacts than normal-spreaders. We assume that the infection probability per contact is the same for both spreader types.

### ODE model

We used the *SEIR* model with susceptibles (*S*), exposed (*E*), infectious (*I*) and resolved (*R*) as possible states to compare our simulated data with an ODE model as shown in Eq. (1). In the *SEIR* model the transmission from state *S* to *E* is modeled by an infection parameter *a* multiplied by the product of *S* and *I*. This product models the number of contacts between the groups of susceptibles and infectious. Furthermore, the transmission from group *E* to *I* and from group *I* to *R* is modeled by linear decay terms with decay parameters *b* and *c*.

1 $$\begin{aligned} \hspace{6cm} \begin{aligned} {\dot{S}}&= -a \cdot I \cdot S \\ {\dot{E}}&= a \cdot I \cdot S - b \cdot E \\ {\dot{I}}&= b \cdot E - c \cdot I \\ {\dot{R}}&= c \cdot I \end{aligned} \end{aligned}$$

Since the ABM has more states than the *SEIR* model, we had to summarize and scale the simulation results for simulation runs with a population of *N* agents into the states of a normalized *SEIR* model as shown in Eq. (2). We used all infectious states ($I_0, I_1, I_2, H$) of our ABM as infectious in the SEIR model and all resolved states of our ABM (*R*, *D*) as resolved in the SEIR model.

2 $$\begin{aligned} \hspace{6cm} \begin{aligned} {\tilde{S}}^{sim}&= \frac{1}{N} S^{sim}\\ {\tilde{E}}^{sim}&= \frac{1}{N} E^{sim} \\ {\tilde{I}}^{sim}&= \frac{1}{N} (I_0^{sim}+I_1^{sim}+I_2^{sim}+H^{sim}) \\ {\tilde{R}}^{sim}&= \frac{1}{N} (R^{sim} + D^{sim}) \\ \end{aligned} \end{aligned}$$

### ABM model

#### Construction of places and agents

The implemented ABM to predict the spread of COVID-19 consists of a set of autonomous agents with given properties, which can move to and stay at different places and have contacts with other agents. This has many similarities to Monte Carlo approaches^44^ but was implemented as an ABM. The contact partners are chosen randomly among the agents at the same place. We created for our ABM 100,000 agents simulating a smaller city and repeated the simulations for each set of parameters 10 times by using different random seeds. To not have too varying results and to ensure that we always have the same starting conditions for lockdown scenarios, we always started with the same initialization of agents and places and used a standardized initiation phase of $\sim 38$ days for all simulations. This means that the random seeds only have an influence from day 39 on. As types of places in our abstract city model we implemented ’households’, ‘schools’, ‘hospitals’, ‘public places’, ’primary care places’ and ’other working places’. By using data from the Statistisches Bundesamt, we adjusted the approximated amount of specific types of places (schools^45^, households^46^ and hospitals^47^). For ’primary care places’ we included.

which would yield approximately 320,000 primary care places for whole Germany. Since some other stores like book stores and flower shops were also partially opened during the lockdown and on the other hand there is some overlap for the places mentioned in Table 2, we estimated that we have one primary care place per 250 agents, which is equivalent to 332,000 primary care places for whole Germany. Additionally, we estimated the amount of ’public places’ and ’other working places’ (see Table 3).

**Table 2:** Branches included in primary care places (column 1) and amount of locations in Germany (column 2) with source references.

Primary care place	Amount in whole Germany
Food stores	34,947^48^
Drug stores	18,753^49^
Post branch	47,000 (26,000 DHL + Hermes)
Gas stations	14,459^50^
Bakeries	45,000^51^
Kiosks	40,500^52^
Doctor’s offices	101,932^53^
Optician branch	11,370^54^
Hearing care	6800^55^

**Table 3:** Number of places per agent amount.

Parameter	Value
#hospitals	1 per 43116 agents
#schools + #childcare	1 per 1148 agents
#households	1 per 2 agents
#primary care places	1 per 250 agents
#public places	1 per 100 agents
#other working places	1 per 50 agents

The number of different places (column 1) is based on the number of agents in the ABM (column 2).

The agents were distributed in to the categories ’children’, ’working adults’ and ’retired persons’ based on the general population of Germany with $\sim 83$ million persons and their age distribution with $\sim 13.2$ million persons under 18 years and $\sim 17.5$ million persons above 65 years^46^. Each category is allowed to visit specific places and have different possible working places (Table 4). For adults we allow hospitals, schools, primary care, public places and other working places as possible working places and adjusted the distribution of jobs again based on data from the ’Statistisches Bundesamt’. The number of people working in primary care, public places and other working places had to be approximated (Table 5).

**Table 4:** Mode of agent movement.

Agent type			
Place type	Child	Retired	Adult
Household	H, F	F	H, F
Hospital	–	–	W
School	W	–	W
Primary care	F	F	W, F
Other working place	–	–	W
Public place	F	F	W, F

Each agent gets for each of the modes ’H’ - time at home, ’W’ - time working and ’F’ - free time, a subset of places as possible destinations assigned. If a mode is assigned for a place type, a random sample of these places is used to create the subsets for each agent.

**Table 5:** Proportion of agent type per place type.

Parameter	Value
Fraction children	0.1745
Fraction retired	0.2319
Fraction working adult	0.5936
Working fraction child care	0.0313
Working fraction hospital	0.0200
Working fraction primary care	0.1500
Working fraction public place	0.2000
Working fraction other working place	0.5987

The distribution of agents per type in the ABM (upper table) and the distribution of working places of working adults (lower table).

For connections between places we defined that places of the types ‘schools’, ‘hospitals’, ‘public places’, ’primary care places’ and ’other working places’ are in principle reachable by all households in both directions. For the households we modeled friendship relationships between households by embedding the households of agents in a relaxed caveman graph^56^. Starting with a cluster of fully connected nodes representing the households, each link is randomly rewired or not with a given probability to end in an average of 7 befriended households.

The created network is afterwards used to assign now agents to places. The majority of agents, except for retired ones, get assigned one household as their home. Additionally, each agent in a household gets all directly connected households assigned as ’befriended households’, which indicates that these households might be selected as a destination for the movement of an agent. Each agent has one household assigned as home and one of the places ‘school’, ‘working place’, ‘hospital’ or ‘primary care’ as working place defined by the agent type (Table 4). Furthermore, each agent selects 4 out of the 7 befriended households, 5 public places and 3 primary care places to visit regularly.

#### Actions of agents

Instead of assigning fixed daily schedules for each agent, we use a randomized approach to select the destination of movements and the length of stay at one place. Whenever an agent moves, one of the three states will be chosen: (i) free time (’F’), (ii) work (’W’) or (iii) home (’H’). Each agent type has for each destination type (’H’,’W’,’F’) an individual subset of possible types of places (Table 4) and furthermore an individualized subset of places as possibilities. For retired agents the exception is that in the case of destination type ‘W’ the destination type ‘H’ is chosen automatically. After the destination type the length of stay is determined based on the mode, using a logistic distribution with a mean depending on the mode (see Table 6). The average time per agent spending at home is set to 12 hours per day^57^ and we assumed an average working time per week of about 42 hours. To reflect for example working breaks or the possibility to spend the free time at more than one place per day, we cut those numbers in half. The current state of an agent based on the infection model influences the decision making for movement destinations. If an agent is in state *H*, the agent has to stay at the hospital and if he is in state $I_2$ the agent stays at home the whole day but still can get visitors. Only agents in state *S*, *E*, $I_0$, $I_1$ or *R* can move freely, whereas agents in state *D* are removed from the system. If under specific lockdown scenarios places like ‘other working places’ or ‘public places’ are not available, the agent goes home, which also reflects the situation of home office, but there is still the possibility of infection within the household.

**Table 6:** Agent length of stay. Average spend time at home, work or for free time. The length of stay at each place is modeled with a standard logistic distribution with given mean value.

Parameter	Value
Mean time at home	6 hours
Mean time at work	3 hours
Mean time free time	3 hours

During the stay at a specific place, each agent has up to $\alpha$ contacts per hour if the agent is a multi-spreader and otherwise up to 1. We propose an unilateral contact model to model the spread of the virus^24^. Therefore, each normal agent has contact to one randomly chosen agent from the same place per hour. Unilateral contact means, that if agent *A* chooses agent *B* to have contact with, *A* might infect *B* (if *A* is infectious and *B* is susceptible), but on the other hand *B* does not automatically has a contact to *A*, unless *B* also chooses *A* as a contact partner. Multi-spreaders choose instead of one contact partner a randomly chosen subset of agents from the same place, up to an estimated maximum number $\alpha$ of contact partners per hour. We estimated $\alpha$ so that the multi-spreaders are responsible for $80\%$ of all infections (Online Resource 1; Supp. Fig. 1).

For each contact between an infectious and a susceptible agent there is a probability *p*, that the susceptible agent gets infected and consequently change his state to *E*. We estimated *p* to fulfill a basic reproduction number $R_0 = 3$ (Online Resource 1; Supp. Fig. 2), which has been shown to be a reasonable number for SARS-CoV-2 in Germany and is in general dependent on a lot of factors such as population density, weather or behavior of the population^39,58,59^.

As a special feature in the ABM, we implemented events as a specific action of the agents. Events are gatherings of agents at one randomly chosen ‘public place’ and are following four specific parameters:

(i) frequency (every 1 to 7 days),
(ii) size (1–10$\%$ of the agents),
(iii) duration (1–10 hours) and
(iv) composition (0–100$\%$ reappearing attendees).

As a standard event we defined the event to take place (i) once a week with (ii) $5\%$ of the population (5000 of 100,000 agents) who are joining for a (iii) 4 hours (h) and in general (iv) no fixed amount ($0\%$) of reappearing participants is expected.

#### Mitigation measures represented in the ABM

To investigate the effectiveness of mitigation measures, we implemented factors like wearing masks and keeping distance for the ABM. As it was shown by Kai and co-workers^60^ performing simulations, masking and in general social distancing measures play a key role for preventing a further spreading of SARS-CoV-2. Our ABM allows the definition of places where social distancing (keeping distance + wearing masks) is required. During the lockdown our ABM was adjusted, so that agents wear masks and keeping distance everywhere except of households. In the ABM the combination of wearing a mask and keeping distance is represented by a single parameter $\kappa$. This parameter is reducing the probability for infecting other agents but the probability of getting infected stays the same. We estimated the parameter $\kappa$ assuming that the decrease of infections during the first lockdown in Germany from March until May 2020 is mainly reflected by these mitigation measures and an intermediate lockdown scenario. As the infection numbers show some delay to the actual infection numbers in Germany, we used the time period from 31.3.2020 ($t=1$) to 18.5.2020 ($t=49$) to fit our model to the observed data. This period of 49 days showed a steady decrease in infection numbers. We scaled and shifted the infection numbers of our simulations, so that the peak of the first wave of infections in Germany is the same as the infection numbers in our simulations when the lockdown is initiated. Here we used a least squares approach to determine which $\kappa$ is the optimal choice. For the least squares approach we transformed the (hourly) infection numbers in our simulations to daily infection numbers, as the infection numbers in Germany were also provided on a daily basis. Let $I_{sim}^{\kappa } (t)$ be the observed simulation results for a given $\kappa$ and day *t* and $I_{Germany}(t)$ the observed daily infection numbers in Germany and let ${\tilde{I}}_{sim}^{\kappa }$ the scaled and shifted version of $I_{sim}^{\kappa }$ as described before. Then we choose $\kappa$ so that

$$\begin{aligned} \hspace{5.2cm} \sum _{t=1}^{49} \left( {\tilde{I}}_{sim}^{\kappa } (t) - I_{Germany} (t) \right) ^2 \rightarrow min \end{aligned}$$

is fulfilled and were able to determine an optimal value of $\kappa = 48 \%$ (see Online Resource 1; Supp. Fig. 3).

As in many countries specific places were closed during the lockdown we implemented the ABM to allow the opening and closing of types of places to test several lockdown scenarios. Agents in the ABM who attempt to visit closed places will return home instead. This procedure will lead to a reduction of contacts as the agents spend more time at home. For all lockdown scenarios we limit the number of visitable befriended households to one fixed household as it was performed in Germany during the first lockdown. Additionally, households with at least one agent in state $I_2$ (symptomatic case) are quarantined and cannot be visited or go out during this state. In our approach we investigated the different lockdown scenarios and compared it to no lockdown as control: (I) Only social distancing measures (wearing masks + keeping distance) but nothing closed; (II) closing ‘schools’, ‘other working places’ and ‘public places’; (III) closing ‘schools’, ‘other working places’ and ‘public places’ and ’primary care’.

### Statistical analysis

For most of the simulation runs, we did 10 replicates with the same starting parameters and initialized different random seeds at the start of the lockdown phase. This ensures the exact same starting behavior for the pre-lockdown phase, allowing for a better comparison of the effects of the lockdown. In Fig. 6 the influence of the single event parameters on the pandemic course is shown. Therefore we investigated how many days it takes for different event parameter settings and lockdown scenarios to get $5\%$ of the population infected. We performed an one-way ANOVA test as provided by the Python module *scipy*.*stats* to find significant changes for varying event parameters or lockdown scenarios. Afterwards we performed for significant outcomes of the ANOVA with a p-value below 0.05 a pairwise tukey posthoc test by using the Python module *statsmodel*.*stats*.*multicomp*. As the ANOVA is not capable of defining which pairwise comparisons are significantly different, we must perform post-hoc pairwise testing and using multi-hypothesis testing correction to analyze pairwise differences. As we define the two samples as independent, we used the Tukey post-hoc approach.

### Comparison of the ABM to different locations in Germany

For all simulation runs of our ABM we used a population size of 100,000 agents. To compare our results to real-world data we chose urban districts and counties of Germany with a population of about 100,000 (see Online Resource 1; Supp. Table 1). For comparison of real world data to our simulated data we shifted the curves of the infection numbers we observed in our simulations, so that the initiation of the lockdown phase fits to the peak of infection numbers of the first wave in Germany in Spring 2020.

## Results

### ABM can be used to simulate lockdown scenarios and their effectiveness

**Figure 2:**
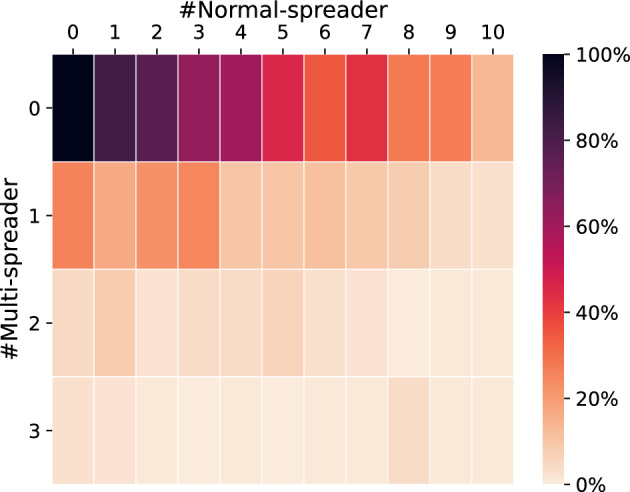
The extinction probability (heat color coded 0% to 100%) is given for the combination of multi-spreader (row) and normal spreader (column) at the begin of the simulation. Probabilities were estimated based on 100 simulation runs per parameter setting.

The modeling of infectious diseases can be conducted using either ODEs or ABMs. In this study, we sought to demonstrate how lockdown scenarios and specifically local events with assigned parameters such as size, duration or frequency affect the spread of the virus. To this end, we employed an ABM to map such changes via simulations. Furthermore, the model was designed to be used at the outset of a pandemic, with reinfections and agents not following specific rules during the lockdown scenario being disregarded. Following the findings of Tellier on the spreading of SARS-CoV-2 via aerosols^61^, we decided to use an asymmetric contact model in our ABM, given the fact that aerosols are not in direct bilateral contact for an infection via the air. To show in the beginning the comparability of our simulations to ODEs and real-world examples from Germany, we had to define a starting amount of infected agents split in multi- and normal-spreaders (Fig. 2). As a preliminary step, we compared the behavior of our ABM by initializing a city with 100,000 agents from whom 1 to 13 have been initially infected and a maximum of 3 of them are by our definition multi-spreader. An investigation of the simulated onset of the pandemic and the subsequent lockdown or early extinction of the virus revealed that 83% of extinction events occurred with 1 normal-spreader, while only 26% of extinction runs could be observed with 1 multi-spreader. With an initial setting of 2 multi-spreaders and 8 normal-spreaders from 100 simulation runs no extinction event occurred (Fig. 2).

**Figure 3:**
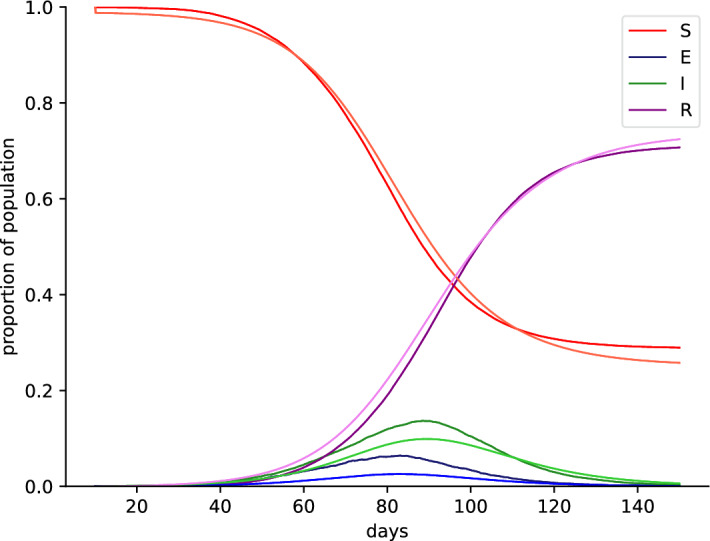
Fitted ordinary differential equation model (Susceptible-Exposed-Infected-Recovered) to observed data from the ABM. ABM states were transformed into SEIR states according to Eq. (2). Light dashed lines are representing the ODE states of SEIR and the corresponding dark solid lines represent the transformed ABM states (light red, red: S; light purple, purple: E; light green, green: I; light blue, blue: R).

In order to guarantee the different behavior of our ABMs without the extinction of the virus (which has not been observed in the real world during the COVID-19 pandemic), we have decided to use a size of 10 infected people, including two multi-spreaders, four our further simulations and analysis. Based on this initial setting we create predictions based on differing random seeds to simulate diverse behaviors in the ABM. Subsequently, the influence of lockdown scenarios, specific parameters within the mitigation measures and also the individual risk for agents related to a specific mitigation measure will be investigated. As the next step, the extended SEIR model in the ABM will be compared to a standard SEIR model as an ODE. Initially, the ABM model of 100,000 agents will be summarised and scaled to the SEIR states of an ODE (Fig. 3). By using a least squares approach, we identified an optimal fit for the parameters *a*, *b*, *c*, $S_0$, $E_0$, $I_0$ and $R_0$ (see section "ODE Model").

**Figure 4:**
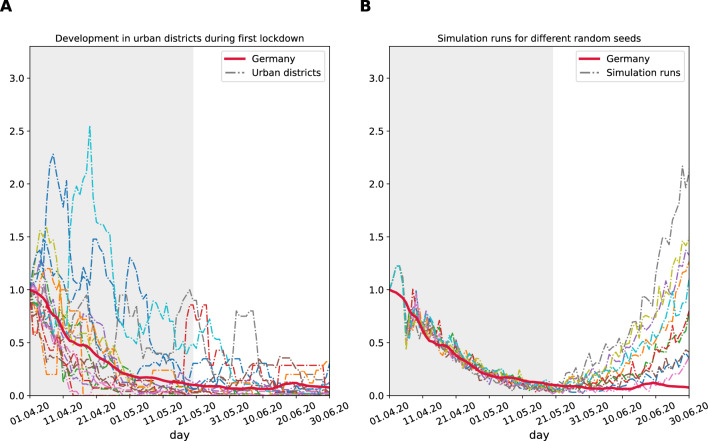
(**A**) Development of infection numbers in relation to the peak of the first wave. For all urban districts the date of the peak for whole Germany were chosen. (**B**) Simulation runs for different random seeds. The social distancing parameter $\kappa$ was used to fit the simulation data to observed infections in Germany.

The comparison indicates that a similar course of the COVID-19 pandemic can be modeled as an ODE and ABM for the individual components susceptible (S), exposed (E), infectious (I) and resolved/recovered (R). The greatest discrepancies were observed for the intermediate states E and I (Fig. 3). Having demonstrated that our ABM exhibits omparable behavior to the ODE employed in numerous publications to forecast the spread of the COVID-19 pandemic^62^, we proceeded to assess the suitability of our model, which incorporates numerous parameters specific to Germany (e.g. agent classes, number of places), for extrapolation to the entire country (Fig. 4, red line). The model was evaluated against counties and urban districts of similar population size (100,000 inhabitants) during the first lockdown phase of 49 days (31.03.2020 to 18.05.2020). The 49 days were set shortly after the official lockdown in Germany (22.03.2020) to account for temporal shifts between the initiation of the lockdown and positive SARS-CoV-2 testing. To ensure comparability with the initial lockdown phase, the lockdown scenarios were concluded on 18.05.2020, resulting in a seven-week lockdown.

In contrast to the average development of the entire country of Germany, the behavior of the individual urban districts demonstrated a onsiderable degree of variation in their relative infection numbers in comparison to the beginning of the lockdown. Interestingly, using our ABM and simulating 10 COVID-19 scenarios with different reinitialized random seeds after starting the lockdown, we see less variance in comparison to whole Germany (Fig. 4B). This leads to the assumption that our agents, despite their stochastic behavior, exhibit more similar patterns based on the settings, resulting in a reduced bias in the analysis compared to real-world citizens of distinct urban districts. As a second observation, in all of our simulated ABM scenarios, the number of infections increased as soon as the lockdown ended, leading to a second wave in June. In contrast, the urban districts and whole Germany remained constant for some time, with the second wave starting later in September 2020. This phenomenon can be explained by the complete relaxation of mitigation measures (wearing masks, keeping distances, etc.) in our simulations in comparison to a partial relaxation in Germany and different urban districts.

### Mitigation measures and specific parameter of events show effect in the infection rate

**Figure 5:**
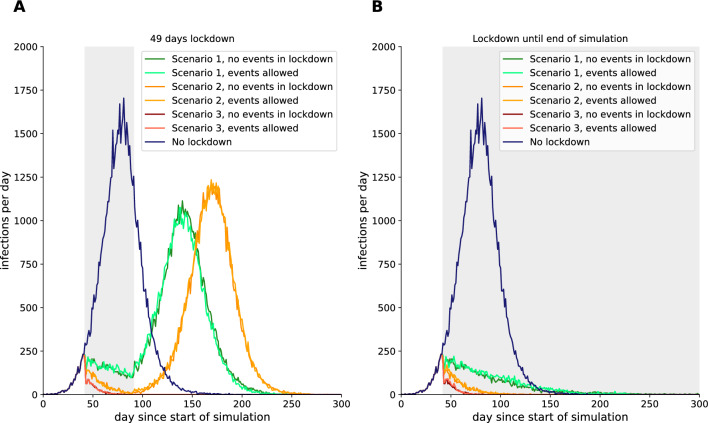
Infections per day for all mitigation scenarios for 49 days lockdown (**A**) and lockdown until the end of the simulation (**B**). Grey shaded regions mean, that a lockdown scenario is active.

In order to focus on the period of the lockdown and the effectiveness of different scenarios, we proceeded to create three different scenarios in principle. These included (i) only social distancing measures but all places are available, (ii) only primary care is open and (iii) every place is closed. In order to assess the impact of different scenarios, we have focused on the effect of permitted and prohibited events. Furthermore, we have compared these results to those obtained in the absence of any lockdown restrictions for a 49-day lockdown (Fig. 5A) or a lockdown until the end of the simulation (Fig. 5B).

In comparison to the scenario of no mitigation measures and no closed places, which demonstrated a peak of infections per day already before day 100, all lockdown scenarios led to a strong reduction in infections per day during the lockdown (Fig. 5A). Furthermore, the losure of specific places had a visible influence on the infection rate. In 40% of our simulations (8 out of 20 runs ), the closure of primary care facilities for the enire lockdown period led to the extinction of the COVID-19 virus.. In the other two lockdown scenarios, i) and ii), a second wave was observed. Here, the looser the lockdown scenario, the earlier the second wave occurred. In addition, the permission or prohibition of our defined standard events (once per week, 4 hours, 5% of agents, no fixed reappearing participants) during the lockdown had no visible influence on the number of infections per day.

In the subsequent step, we investigatedthe impact of varying the parameters of events on the general course of the pandemic. Therefore, we altered the parameters size (1–10% of the agents), duration (1–10 hours), frequency (every 1–7 days one event) and also the composition of fixed reappearing participants (0–100%). In our approach, we varied one of the parameters while maintaining the remaining parameters at their default alues to assess the direct impact of the change. For comparing the results we analyzed how long it took to infect between 5% or up to 50% of the 100,000 agents in our simulation (Online Resource 1; Supp. Figs. 4–9).

**Figure 6:**
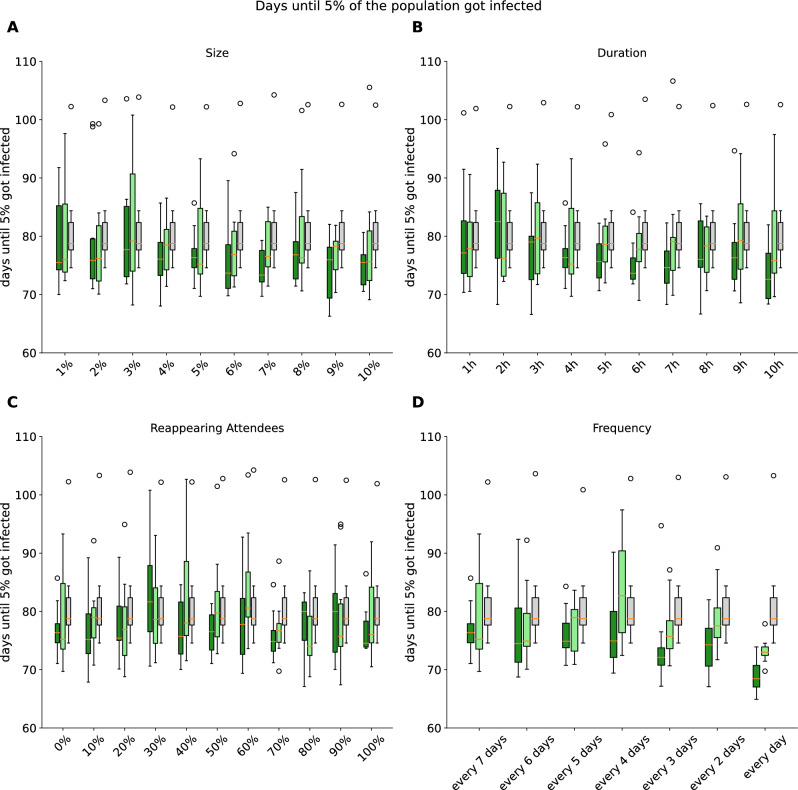
(**A**–**D**) Box-Whisker-Plots represent distribution of amount of days until 5% of agents got infected within 10 simulation runs with different random seeds. (**A**) size of the event, (**B**) time of the event, (**C**) percentage of reappearing attendees, (**D**) frequency of events per week. Coloring of boxplots show the different lockdown scenarios (dark green: events without mask, light green: events with masks, grey: no events).

It was not possible to observe clear dependencies between an increase in the event parameter changes due to size (Fig. 6A), duration (Fig. 6B) or reappearing attendees (Fig. 6C) whether wearing masks (and keeping distance) (light green) or not wearing masks (dark green). In contrast to the other parameters, the frequency of events (Fig. 6D) demonstrated a descending trend, with events occuring once a week to daily events (Fig. 6D). The number of days until 5% of the agents were infected decreased from 76 to under 69. A comparison of the variances within different sizes, durations or reappearing attendees showed no significant differences, regardless of the mask wearing behavior (ANOVA: p-values >0.1). Only within the event frequencies were significant changes observed (wearing masks: p-value = 0.031; without wearing masks: p-value = 0.029). A pairwise Tukey post-hoc test (Online Resource 1; Supp. Fig. 10–13) for events with social distancing revealed significant differences between daily events and events that take place once every four days (adjusted p-value of < 0.01). In the case of events without social distancing (no masks, no additional distance), significant differences were observed between daily events and events that occur once every four days (adjusted p-value of 0.036) and weekly events (adjusted p-value of 0.039). In addition, a direct comparison of scenarios that permit events with and without the use of masks revealed notable differences in the number of days required w to infect 5% of the agents. The results indicated that the impact of masks is more pronounced when the duration and frequency of events are longer and higher, respectively. However, this trend was less evident in the context of event size and the percentage of reappearing attendees.

**Figure 7:**
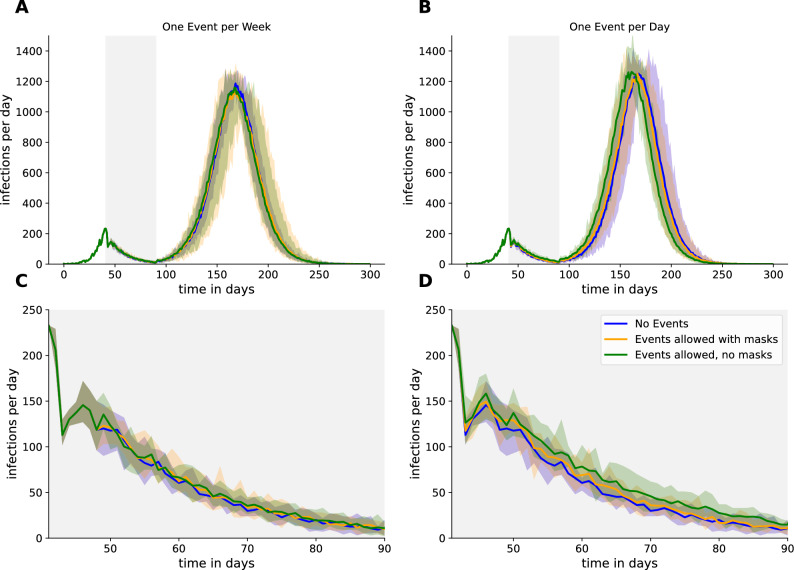
Investigation of infection curves for the event frequency as the parameter with the strongest influence. Lowest ((**A**) and (**C**)) vs Highest ((**B**) and (**D**)) event frequency is shown for the whole simulation run. Lines are showing the mean infection numbers of the 10 simulation runs for each setting. Shaded regions are showing the min/max region for the 10 runs. In (**C**) and (**D**) only the lockdown period is shown.

After getting an overall view of the parameter influence for events we proceeded to examine the infections per day during the full simulation (Fig. 7A,B) and only the lockdown period (Fig. 7C,D). To this end, we employed a comparison of the standard frequency (once per week) to the highest frequency (every day). The area plots demonstrate that an earlier second wave emerges in the scenario of unrestricted events in all simulated runs (Fig. 7B). When focusing only on the lockdown period, the scenarios that permitted events with and without social distancing measures demonstrated a consistent prevalence above the scenario that did not permit events (Fig. 7D).

### Individualized infection risk on events

**Figure 8:**
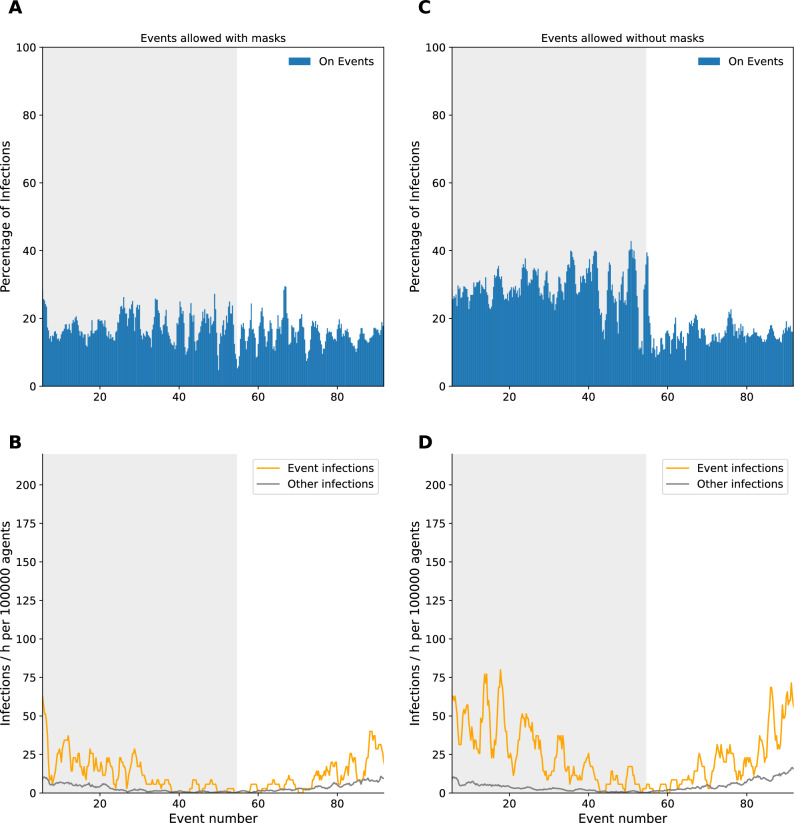
Proportion of infections caused on events compared to infections occuring at the same time offside of events for (**A**) events allowed during lockdown with masks and (**C**) events allowed during lockdown even without masks. Infections per hour were compared by measuring infections per 100,000 agents in (**B**) and (**D**).

These results on a daily event basis showed that the infection probability is affected by the event. However, it is possible that the overall infection process within a week may mask this effect. Consequently, the subsequent analysis focused on the specific points in time where events were taking place and correlated the event infections with the total infections (Fig. 8). During the time an event takes place $\sim 16.8\%$ (caring about social distancing) or $\sim 29.4\%$ (not caring about social distancing) of all infections are related to the event.

The results of our simulations indicate that the implementation of mitigation measures on events has a major impact on the number of infections. The number of infections on events without social distancing was approximately twice as high as on events with social distancing (Fig. 8). If now the $\sim 16.8\%$ / $\sim 29.4\%$ of infections during an event is set in relation to the lower number of agents (only $5\%$) who can be affected as participants of an event, we see a more drastic effect of the events on the single participant (Fig. 8B). Here in peak during the lockdown $\sim 25$/$\sim 50$ infections per hour and 100,000 agents could be observed. In contrast, the number of infections not related to events was around 10 per hour and 100,000 agents. This clearly shows a higher risk for agents attending events during the COVID-19 pandemic. Besides, we observed some kind of wave pattern for the infection numbers on consecutive events. As infected agents must go into quarantine, new agents are added to events to maintain their size. By doing so, on events with a lot of infected agents follows a phase with lots of quarantined (and thus replaced) agents, which makes following events to start with less infected.

**Figure 9:**
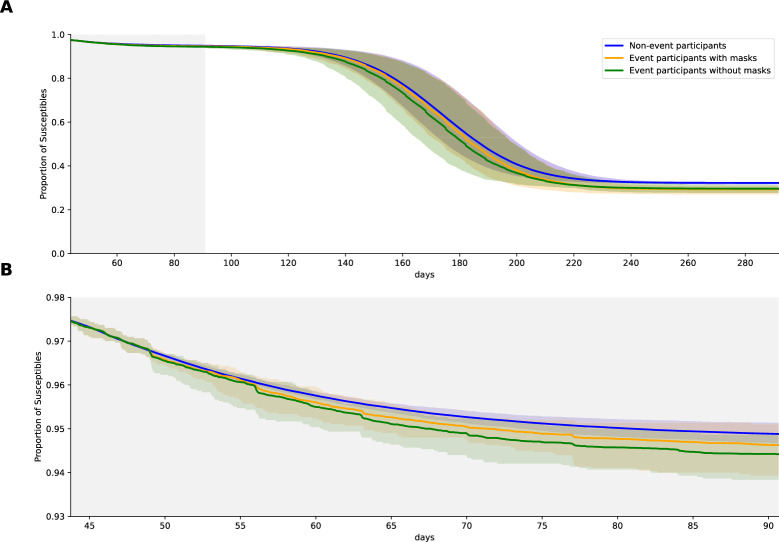
Proportion of susceptibles for non-event participants, event participants with masks and event participants without masks for the whole time period (**A**) and for the lockdown period (**B**).

During the lockdown period, the proportion of susceptible agents for non-event participants followed a hyperbolic curve. In contrast, for event participants, independent of their level of concern about social distancing, there were clear drops in susceptible agents, which were correlated with the event points in time (Fig. 9B). The difference in the proportion of susceptible agents between event and non-event participants can be transferred to an individual risk increase of 21% for participants of events where everybody is wearing a mask and is keeping distance. The aforementioned increase is even more pronounced (48%) in instances where events are held without the use of masks or adherence to social distancing guidelines. This is in comparison to the complete prohibition of events during the lockdown period (Fig. 9B).

**Figure 10:**
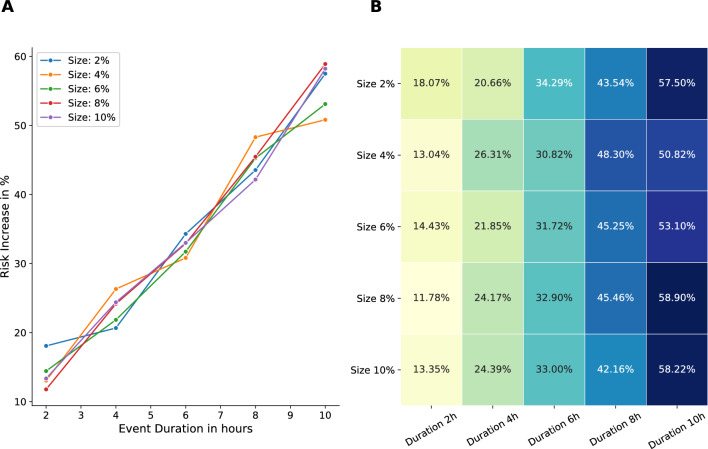
Increase of individual risk for reappearing attendees (wearing masks) compared to non-event participants during the lockdown. Size and Duration were varied, all other parameters were kept as in our standard event setting.

Another area of interest was the increase in the individual risk of infection for reappearing attendees of events during the lockdown period (Fig. 10), with regard to the size and duration of the events. Our findings indicated that the individual risk appears to increase linearly with increasing event duration, but remains unchanged for increasing event sizes. The observed increase in risk for longer event durations can be attributed to the fact that the longer an event lasts, the more frequently susceptible agents come into contact with infectious agents. It can be anticipated that the risk will exhibit a lower than linear increase for durations exceeding a certain threshold, as there is an upper limit to the number of agents that can be infected. Once all event participants have been infected, there will be no further transmission. The behavior we see for an increase of the event size can be explained by the upper limit of contacts per hour for an agent. Even at relatively small events, the upper limit is reached, indicating that further increases in event size do not result in increased contact rates per hour and, consequently, an increased individual risk of infection.

## Discussion

In conclusion, we developed a lightweight stochastic ABM that does not require extensive data sources to analyze the course of the pandemic caused by COVID-19 under different lockdown scenarios. The ABM can consider local influences in greater detail than an ODE model, which is limited in spatial resolution for highly fragmented modeling domains such as cities, with an abstract infrastructure and agent movement pattern^63^. One disadvantage of this approach is that it is unable to simulate the correct number of specific places and to define agent roles in the same way as in ABM models for specific places like Florida^31^ or specific dependencies like resources and factories^34^. Furthermore, it is not capable of simulating small-world networks^33^ or of analyzing the spread of the virus to other cities through human mobility^32^. This model does not focus on pharmaceutical interventions or vaccinations. Also, longer pandemics will be not accurately simulated and we did not consider evolution of the virus or checking the lockdown scenarios and their events on future waves. On the other side allows our approach to get information about an abstract city with flexible and adjustable infrastructures mimicking different cities. Our ABM has the advantage of being easily adaptable with few parameters, making it ideal for making fast and early decisions based on lockdown scenarios. This is a hybrid approach between complex ABMs and abstract ODE models estimating upper bounds for policy making effects in the start of a pandemic as well as estimating lower bounds if people start to willingly perform protective actions. The model permitted the investigation of diverse types of places including primary care facilities and agent roles like retired persons, without being restricted to specific communities like Gangelt^24,64^. The advantage is the focus on specific parameters or places like primary care facilities or events and their respective parameters, such as duration, frequency, and reappearing attendees.

The benefit of such an ABM also in relation to the COVID-19 pandemic is based on the implementation of possible interventions like mitigation measures, quarantine routines and lockdown scenarios over specific time periods, as well as health state related behavior changes of agents. In contrast to ODE models, our approach sticks to one abstract flexible ABM to model a standard city of Germany with 100,000 citizens using a fairly small amount of parameters including of places, agent roles and movement options, mitigation measures and disease related values. The comparison of our model to different counties geographically spread across Germany demonstrated its ability to balance out small differences in the initiation of lockdown scenarios and still provide reliable simulations on an abstract level. By this our ABM tries to compensate the huge time consumption increase from high complex ABMs. This is another option than the fitting of ABM parameters in a macroscopic ODE model^1^. In addition, this allows for more easily adjusting the ABM to diverse city infrastructures. Conversely, our approach allows us to gain insight into an abstract city with flexible and adjustable infrastructures that can be adapted to represent different cities. It is a combination of strengths of complex ABMs and abstract ODE models. Our ABM estimates upper and lower bounds for the effects of policy making during the early stages of a pandemic, as well as the effects of people performing protective actions. One main feature is the effect of different lockdown scenarios at the city scale resolution with the inclusion of, for example, primary care places and local events. The closing of primary care places had a big influence on the spread of the virus, as lockdown scenario iii) led to full extinction of COVID-19 in 40% of simulations within a lockdown period of 49 days. As it is not possible to close primary care systems completely, it would be interesting to categorize the primary care system based on need and analyze the influence of average length of agent stay or the crowding in one place to get more information about possible optimal control strategies. In our case we were not focusing on the primary care system but on the effect of local events on the spreading of COVID-19 dependent on putative influential factors such as size, duration, frequency and reappearing attendees. In general, it was not possible to observe cut-offs related to size, duration or reappearing attendees of events for an optimal control strategy. Only the frequency of local events showed a significant increase in the infection rate during the COVID-19 lockdown simulations. In future our ABM approach could be adapted to focus on specific parameter settings to mimic events like soccer games and family parties and compare their infection risk to develop optimized lockdown scenarios. In general, our simplistic ABM in this study can give first impressions to approximate possible outcomes of lockdown scenarios or their relaxations at the beginning of a pandemic.

To the best of our knowledge, this is the first instance of a city scale resolution ABM incorporating the prohibition of events as a specific mitigation intervention to a pandemic course. In these simulations, we demonstrated that different random seeds and replicates are essential to verify the simulations of an ABM, as the effects were not as pronounced as those observed in different counties within a country. However, they still exhibited considerable variance (Fig. 4). This ABM allows without setting specific places based on city maps a first approximation of the influence of lockdown scenarios during a pandemic. Based on the observation of a basic reproduction number ($R_0$)^39,58,59^ we can estimate the infection probability for contacts between agents in our ABM. With this we can compare different mitigation scenarios and assess the individual infection risk for different lockdown scenarios. Addition of a priori information from external experiments like aerosols in indoor places^65^ can even increase the prediction accuracy.

Our ABM showed an increased individual infection risk of reappearing attendees at local events (Fig. 9). Further, this effect was doubled without mitigation measures. In our example of the COVID-19 simulations, we found an 21% / 48% infection risk increase for reappearing attendees (on events with masks and distancing / no masks and no distancing) during the lockdown period. Also, single parameters like duration, size, reappearing attendees and especially frequency of local events have a direct influence on the number of COVID-19 infections (Fig. 6). Furthermore, we observed a linear growth in the individual infection risk in relation to the duration of events, whereas the size of the event showed no influence (Fig. 10). Interestingly, our local events do not appear to be the main driving factor in our lockdown scenarios for the spread of COVID-19. This is in line with the findings of Lee and colleagues, who suggest that most COVID-19 infections are now assumed to have occurred within households^66^. These results focusing on local events during the pandemic showed the influence of events on the individual infection risk under specific event parameter combinations. Interestingly, single parameter variations except of reappearing attendees showed no statistical significance in the overall community. The recent interest in predicting the risk of infection at specific locations for pandemic diseases such as COVID-19 has already been investigated in the context of workplaces^67^. However, there is also interest in local events, as evidenced by this analysis, which aims to influence future policy-making by making decisions more transparent and dynamically adjustable.

### Supplementary Information

Supplementary Information.

## Data Availability

The scripts for generating and analyzing the datasets during the current study are available in the github Covid19_ABM repository https://github.com/Stegobully/Covid19_ABM. The datasets for specific simulation parameter settings including all txt-files of the current study are further available from the corresponding author on reasonable request.
