# Supplementary material for: Agent-based modeling to estimate the impact of lockdown scenarios and events on a pandemic exemplified on SARS-CoV-2
Source: Sci Rep. 2024 Jun 11;14:13391. doi: 10.1038/s41598-024-63795-1 (PMC11167020; doi:10.1038/s41598-024-63795-1)
Supplement: Supplementary file 1 — Supplementary Information. [file 41598_2024_63795_MOESM1_ESM.pdf]

Supplementary Material

Agent-based modeling to estimate the impact of lockdown scenarios and events on a pandemic exemplified on SARS-CoV-2

Published in

Scientific Reports

Authors

Christian Nitzsche and Stefan Simm
University Medicine of Greifswald

Institute of Bioinformatics
University Medicine Greifswald
17475 Greifswald
stefan.simm@uni-greifswald.de

Estimating the Upper Limit α of Contacts for Multispreaders

Normal Spreaders and Multispreaders are distinguished in the model via a different amount of contacts. To ensure that Multispreaders are responsible for 80% of all infections we let them have 80% of all occuring contacts during a simulation, making all contacts the same probability of causing an infection. Multispreaders can have contact to multiple other agents at the same place, limited by a parameter α as the upper limit of possible contacts per timestep. By running simulations for different upper limits of contacts we could identify a value of $\alpha = 72$ as the maximum number of contacts per timestep for multispreaders to ensure that multispreaders are responsible for 80% of all infections.

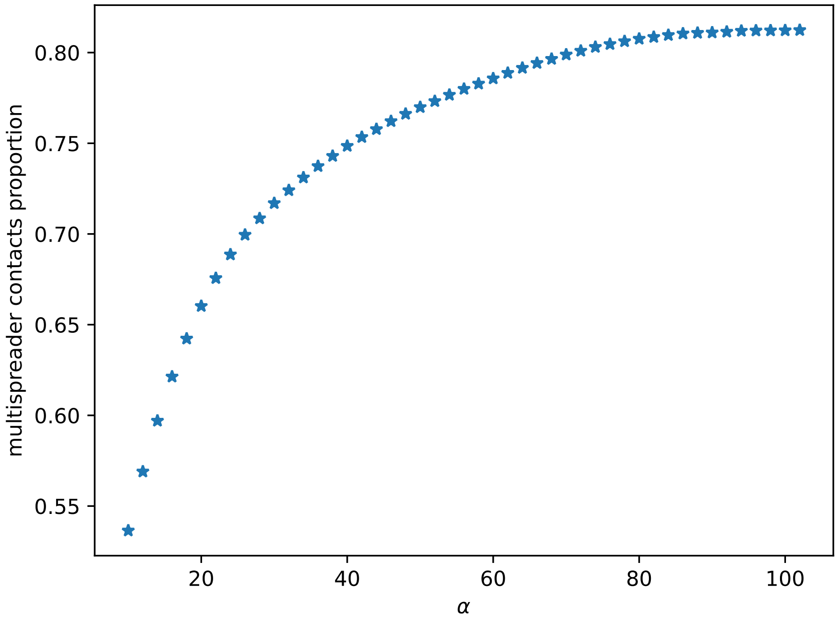

Supp. Figure 1: Proportion of multispreader contacts in dependence of an upper limit α of contacts for multispreaders per timestep.

Estimating the Infection Probability p_0

For estimating the infection probability in our model we ran simulations for different infection probabilities p_0 and used the observed infections to calculate a basis reproductive number R_0 for the pre lockdown phase. We observed a linear relationship (at least for $R_0 \leq 4.5$ and used a linear regression model to obtain a final value for R_0.

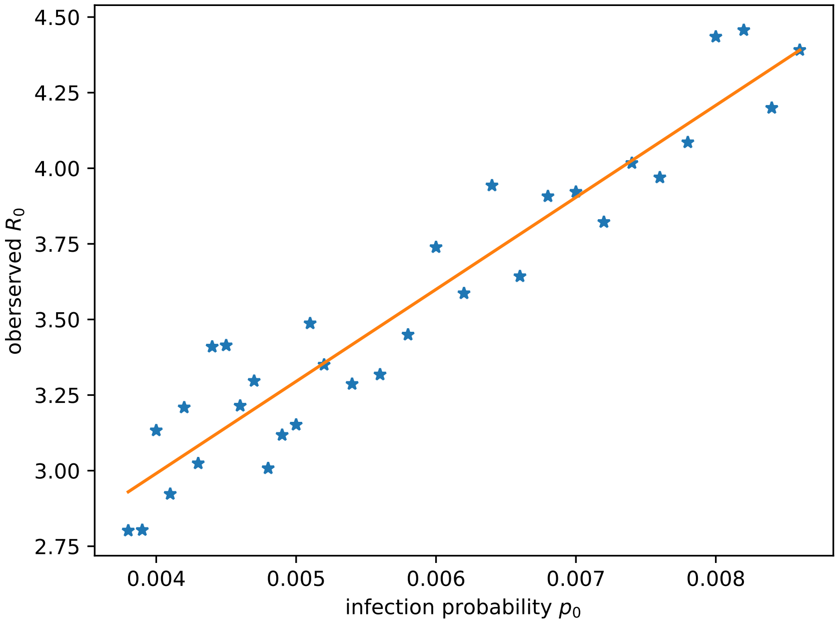

Supp. Figure 2: Observed basis reproductive number R_0 for a given infection probability.

Estimating the Social Distancing Parameter κ

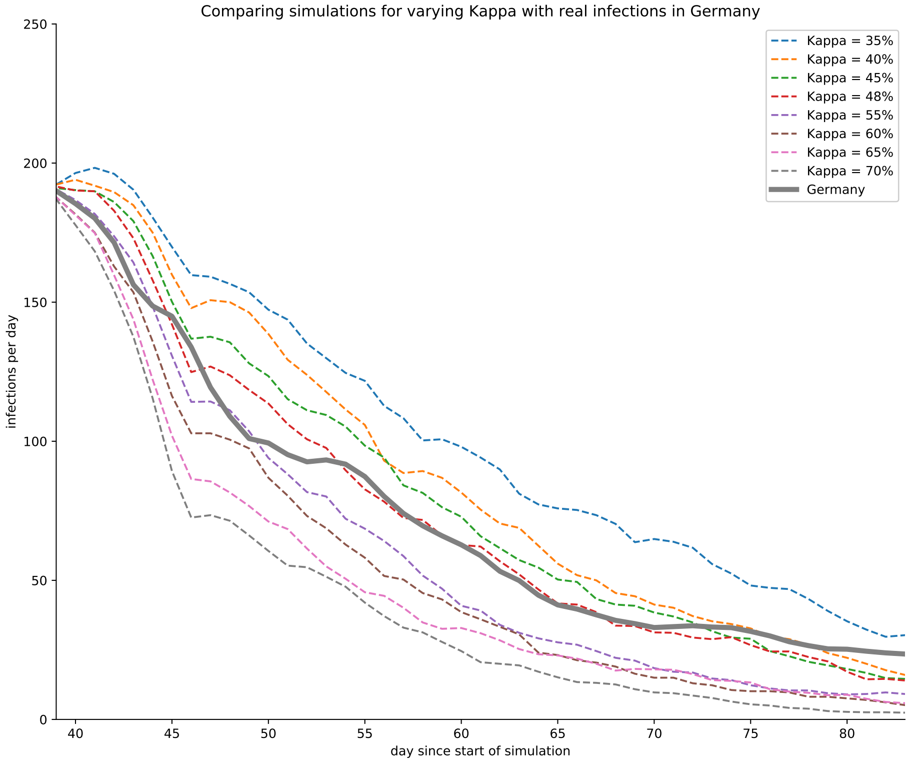

Supp. Figure 3: Least squares approach to estimate an optimal value for κ during the first lockdown period in Germany from March til May 2020.

Urban Districts and Cities

District	Population	District	Population
Bottrop	117.311	Pforzheim	125.529
Cottbus	98.359	Remscheid	111.770
Erlangen	113.292	Salzgitter	103.694
Flensburg	91.113	Schwerin	95.740
Fürth	129.122	Trier	110.570
Heilbronn	125.613	Ulm	126.949
Jena	110.502	Wolfsburg	123.949
Kaiserslautern	99.292	Würzburg	126.933
Koblenz	113.638		

Supp. Table 1: Selection of german urban districts with about 100.000 citizens. Populations are as of 31.12.2021.

Boxplots to visualize how many days are needed until a given percentage of the population get infected

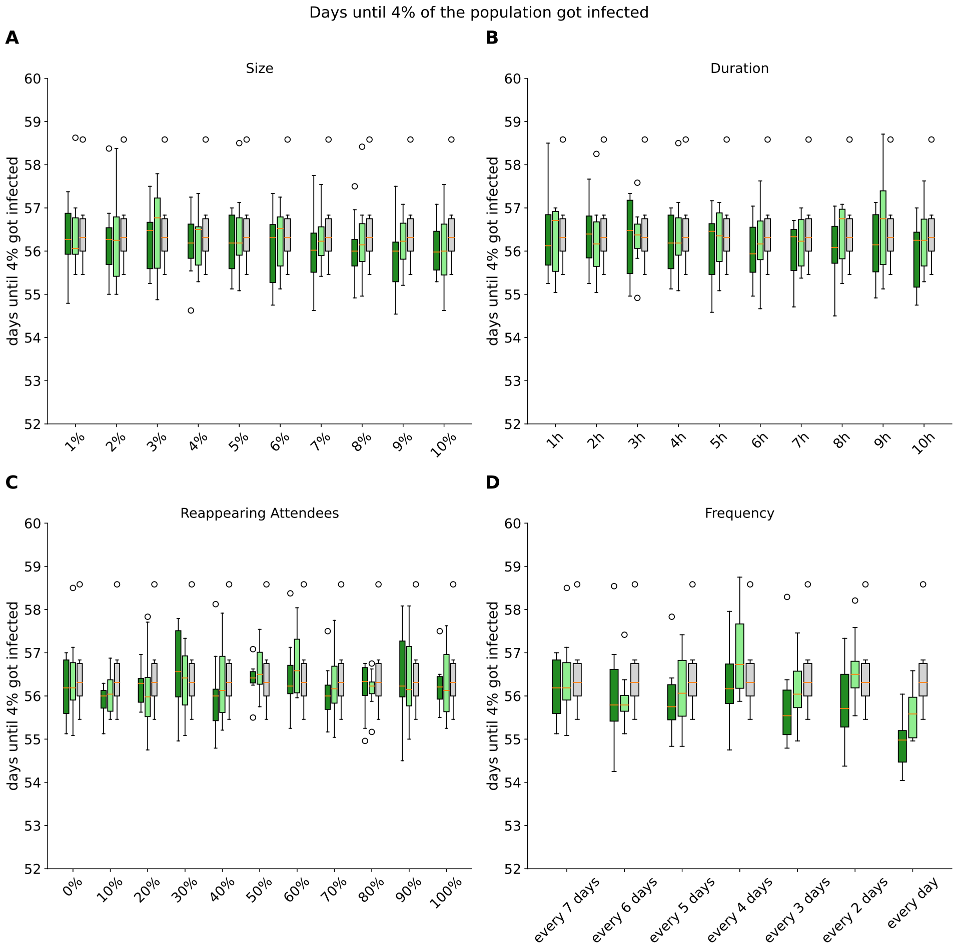

Supp. Figure 4: (A-D) Box-Whisker-Plots represent distribution of amount of days until 4% of agents got infected within 10 simulation runs with different random seeds. (A) size of the event, (B) time of the event, (C) percentage of reappearing attendees, (D) frequency of events per week. Coloring of boxplots show the different lockdown scenarios (dark green: events without mask, light green: events with masks, grey: no events).

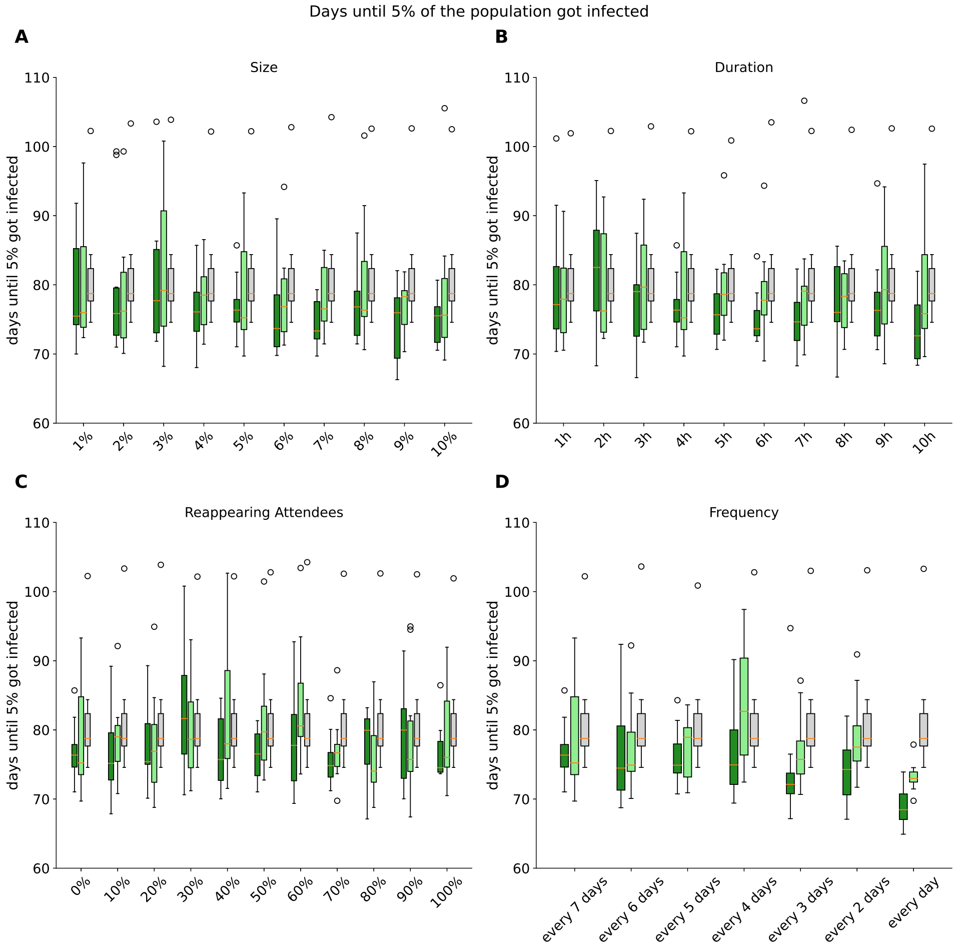

Supp. Figure 5: (A-D) Box-Whisker-Plots represent distribution of amount of days until 5% of agents got infected within 10 simulation runs with different random seeds. (A) size of the event, (B) time of the event, (C) percentage of reappearing attendees, (D) frequency of events per week. Coloring of boxplots show the different lockdown scenarios (dark green: events without mask, light green: events with masks, grey: no events).

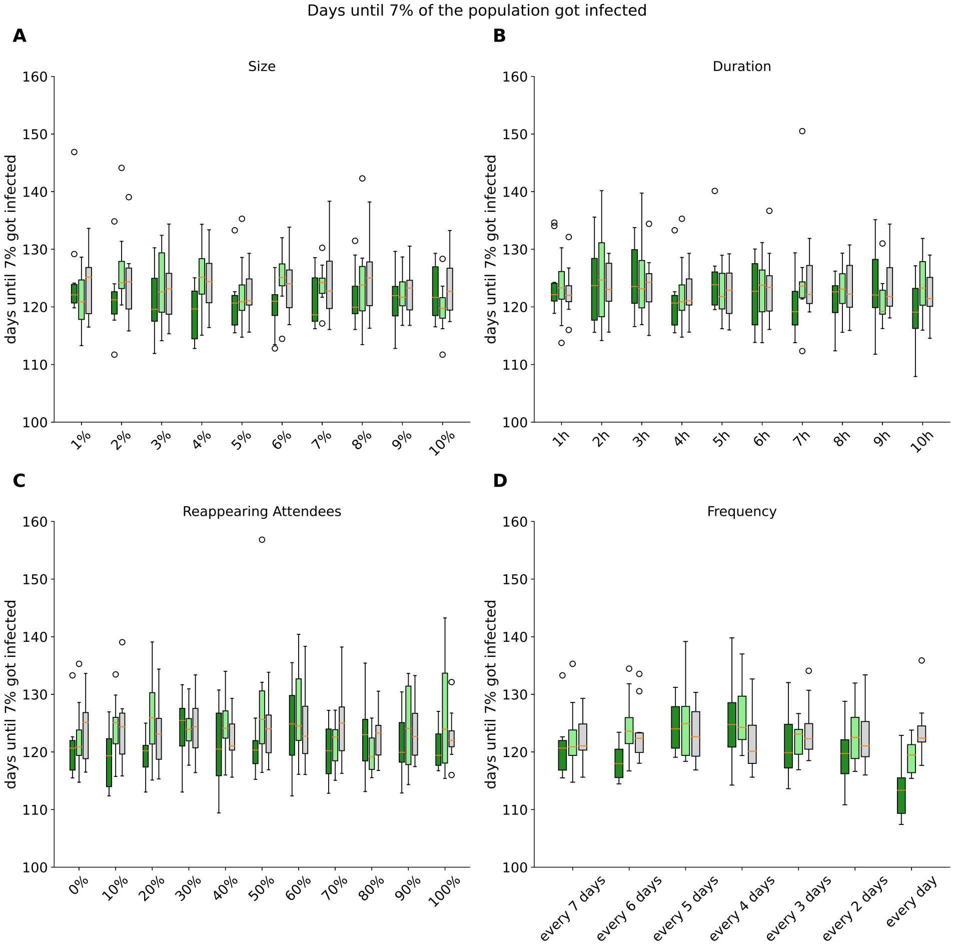

Supp. Figure 6: (A-D) Box-Whisker-Plots represent distribution of amount of days until 7% of agents got infected within 10 simulation runs with different random seeds. (A) size of the event, (B) time of the event, (C) percentage of reappearing attendees, (D) frequency of events per week. Coloring of boxplots show the different lockdown scenarios (dark green: events without mask, light green: events with masks, grey: no events).

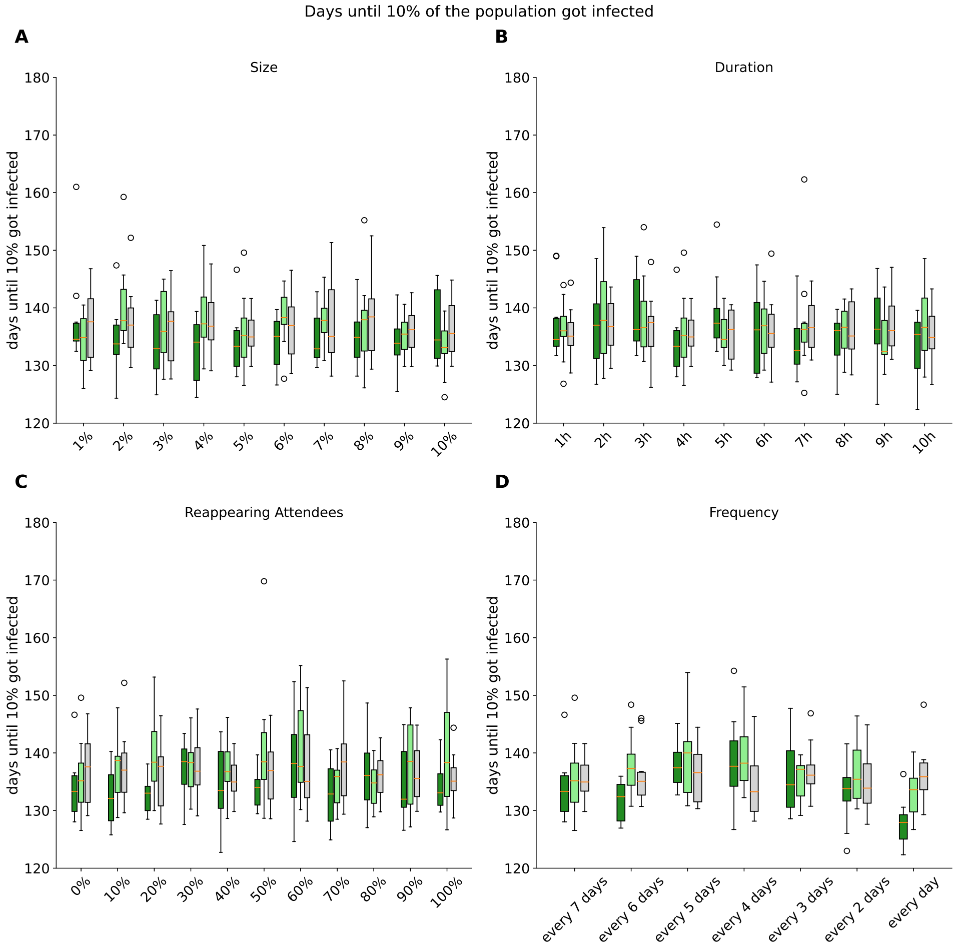

Supp. Figure 7: (A-D) Box-Whisker-Plots represent distribution of amount of days until 10% of agents got infected within 10 simulation runs with different random seeds. (A) size of the event, (B) time of the event, (C) percentage of reappearing attendees, (D) frequency of events per week. Coloring of boxplots show the different lockdown scenarios (dark green: events without mask, light green: events with masks, grey: no events).

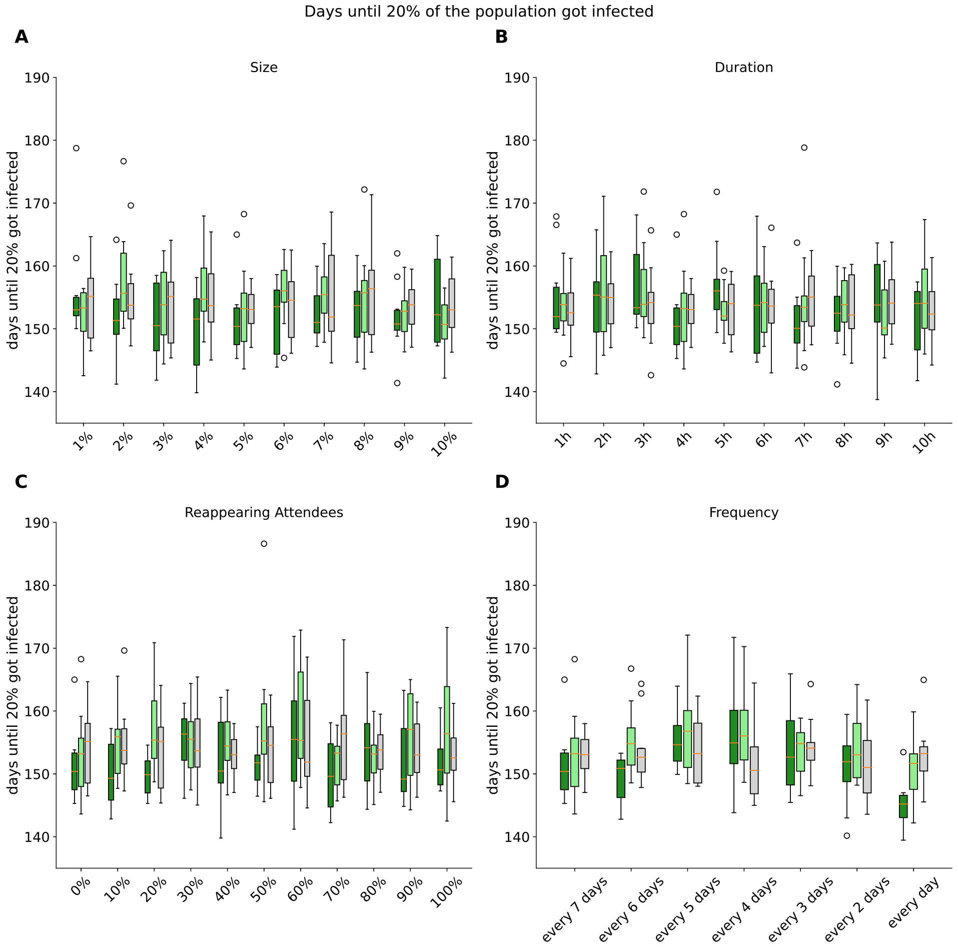

Supp. Figure 8: (A-D) Box-Whisker-Plots represent distribution of amount of days until 20% of agents got infected within 10 simulation runs with different random seeds. (A) size of the event, (B) time of the event, (C) percentage of reappearing attendees, (D) frequency of events per week. Coloring of boxplots show the different lockdown scenarios (dark green: events without mask, light green: events with masks, grey: no events).

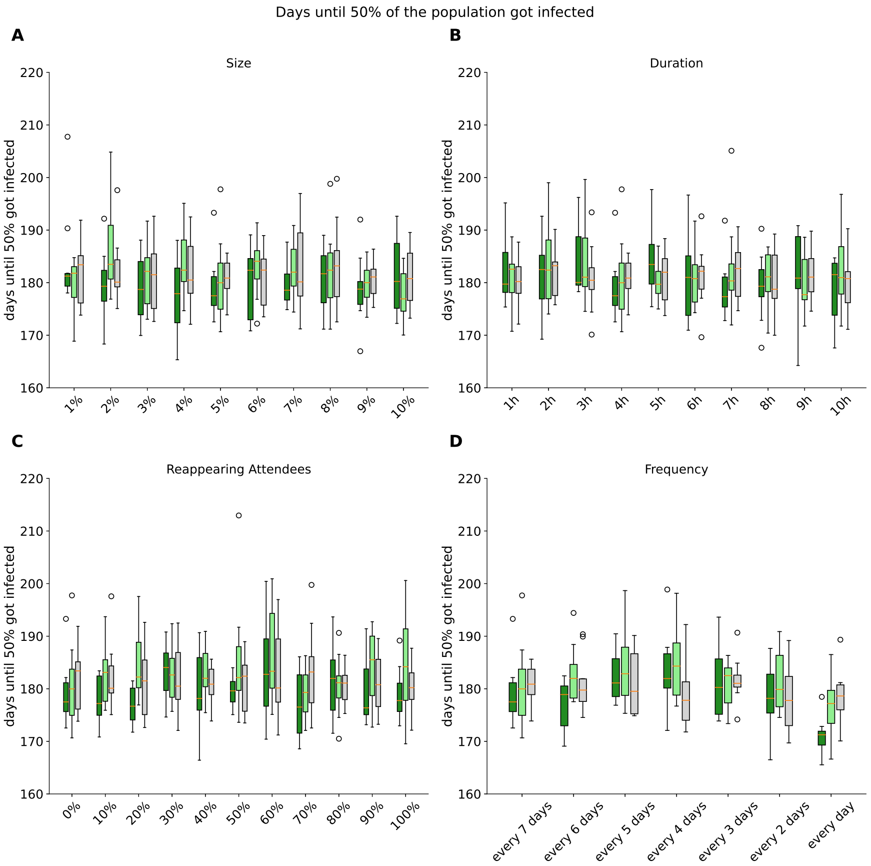

Supp. Figure 9: (A-D) Box-Whisker-Plots represent distribution of amount of days until 50% of agents got infected within 10 simulation runs with different random seeds. (A) size of the event, (B) time of the event, (C) percentage of reappearing attendees, (D) frequency of events per week. Coloring of boxplots show the different lockdown scenarios (dark green: events without mask, light green: events with masks, grey: no events).

Statistical Analysis

We performed an ANOVA test for a fixed masking situation (wearing masks / not wearing masks) and varying the parameters Size, Duration, Composition and Frequency. Afterwards we performed a Tukey post-hoc test for pairwise comparisons for event parameter changes.

Frequency

ANOVA p-value: 0.031 / 0.029 (wearing masks / not wearing masks)

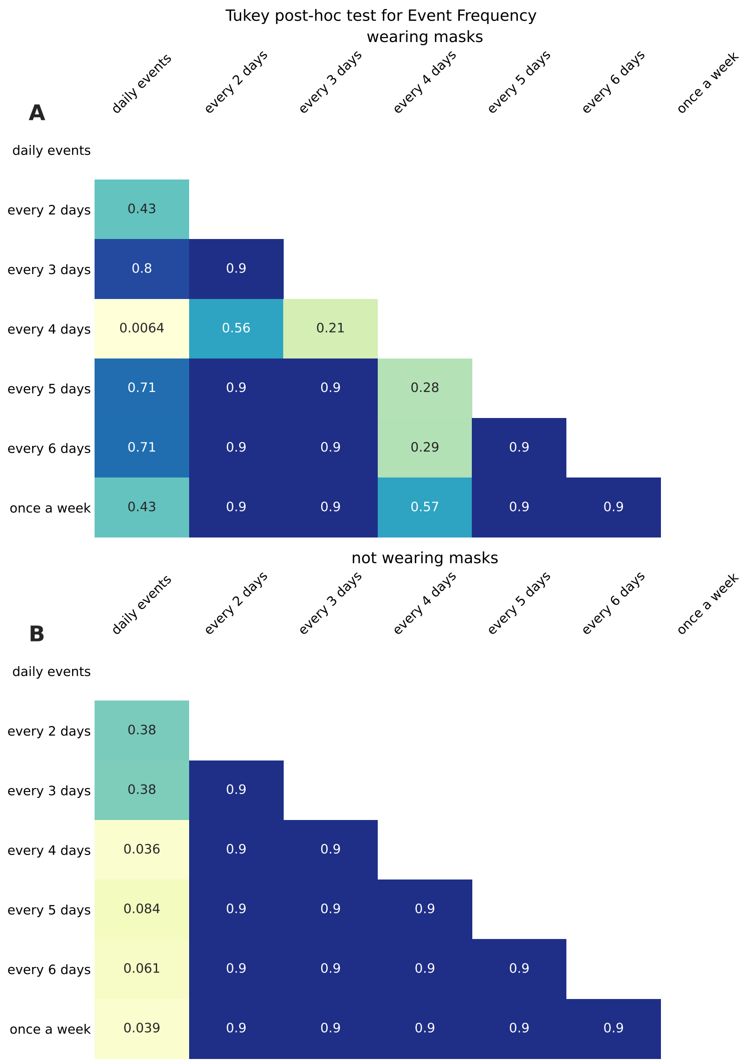

Supp. Figure 10: Pairwise Tukey post-hoc test. Adjusted p-values are shown for the scenarios (A) Event frequency was varied and agents are wearing masks, (B) Event frequency was varied and agents are not wearing masks.

Size

ANOVA p-value: 0.945 / 0.395 (wearing masks / not wearing masks)

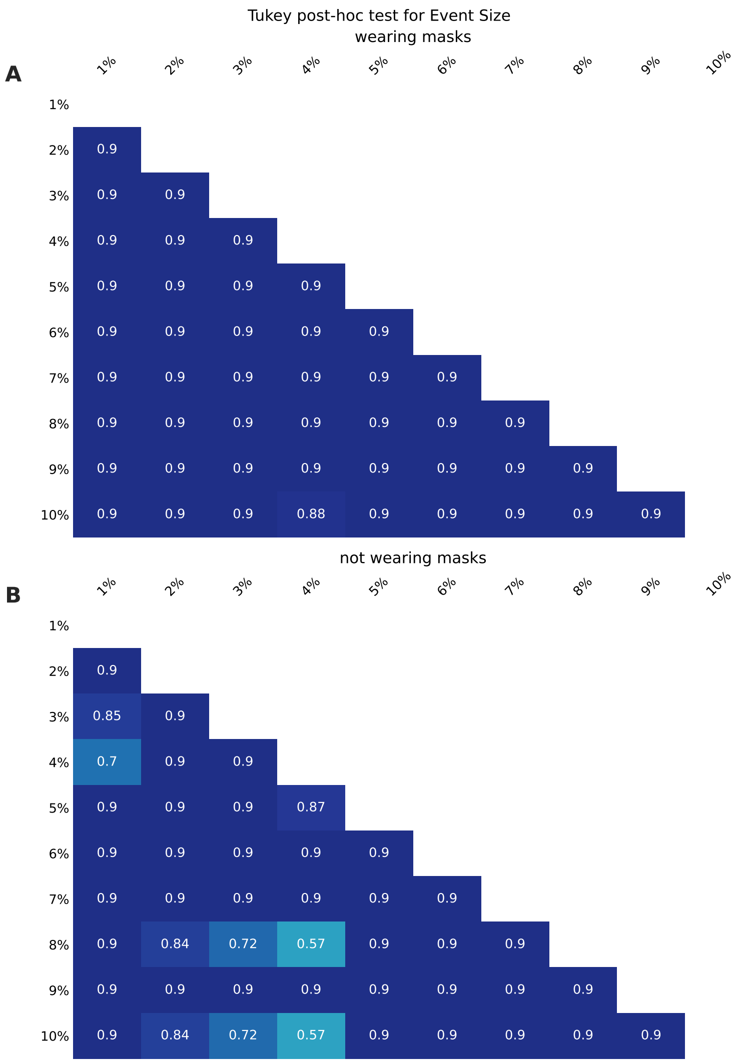

Supp. Figure 11: Pairwise Tukey post-hoc test. Adjusted p-values are shown for the scenarios (A) Event size was varied and agents are wearing masks, (B) Event size was varied and agents are not wearing masks.

Duration

ANOVA p-value: 0.999 / 0.108 (wearing masks / not wearing masks)

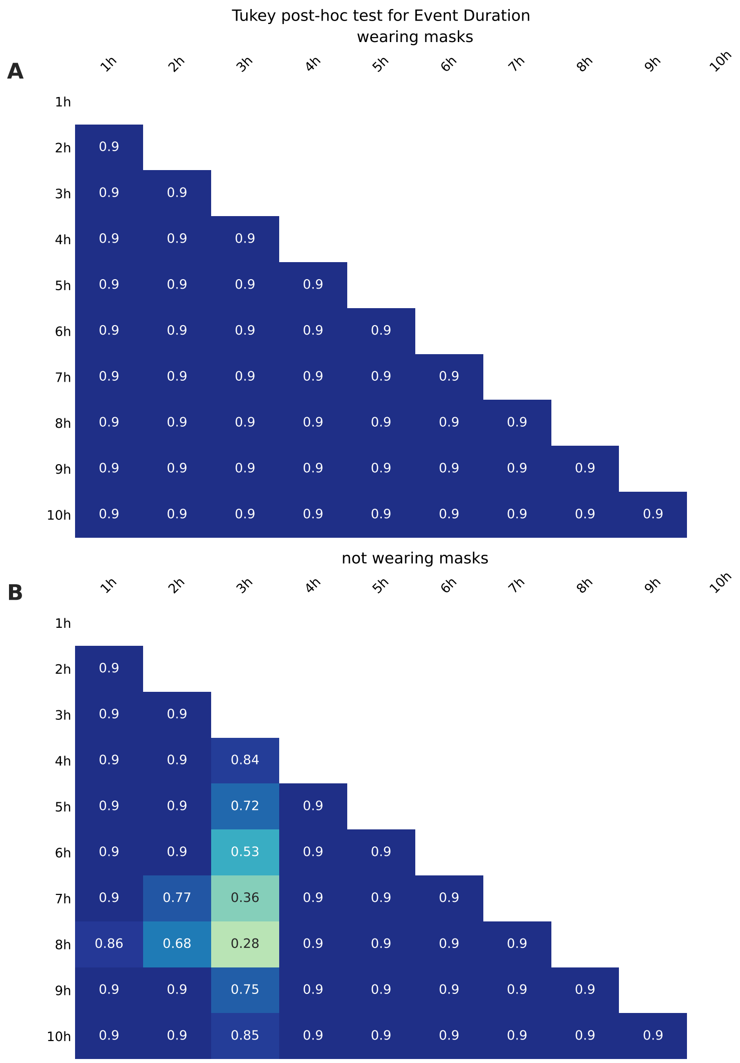

Supp. Figure 12: Pairwise Tukey post-hoc test. Adjusted p-values are shown for the scenarios (A) Event duration was varied and agents are wearing masks, (B) Event duration was varied and agents are not wearing masks.

Composition

ANOVA p-value: 0.649 / 0.397 (wearing masks / not wearing masks)

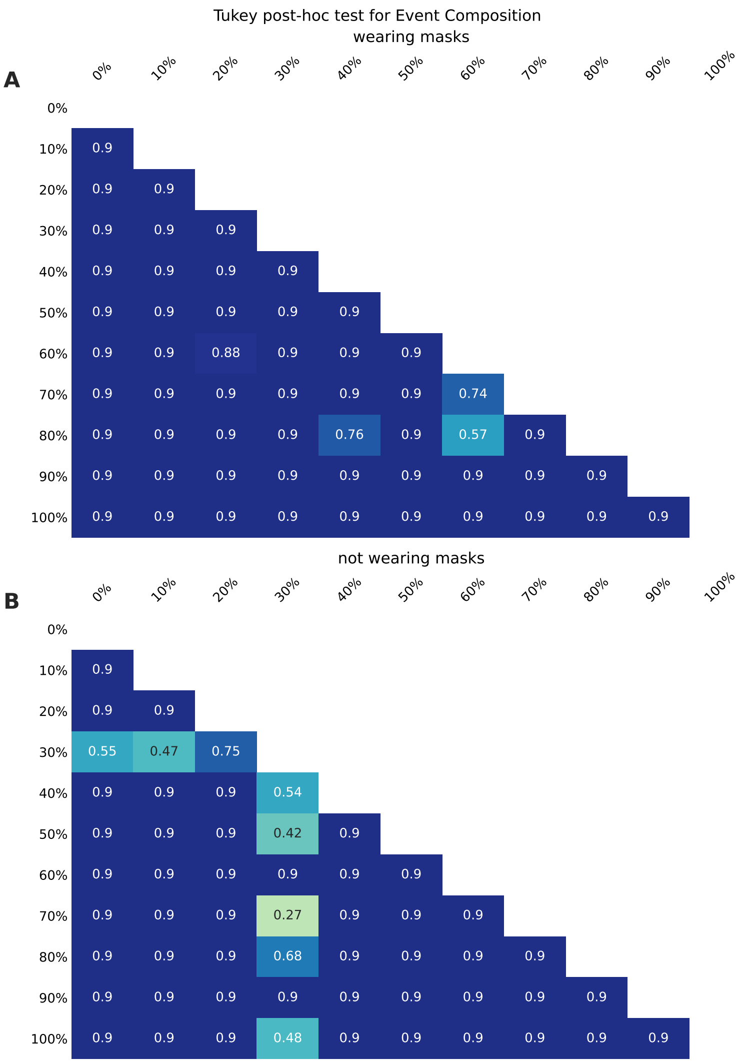

Supp. Figure 13: Pairwise Tukey post-hoc test. Adjusted p-values are shown for the scenarios (A) Proportion of reappearing attendees was varied and agents are wearing masks, (B) Proportion of reappearing attendees was varied and agents are not wearing masks.

Simulation process

Important Classes

Places
place type<br><br>list: of agents currently at this place<br><br>bool: is closed<br><br>list: neighbors within the contact network

Agent
agent type<br>health state<br>place: current position<br>place: working place<br>place: home<br>list: preferred places to visit<br>bool: is wearing mask<br>bool: is multispreader<br>int: time at current position<br>int: time in current health state<br>bool: is in quarantine<br>bool: is event participant

Initialization
create agents;
create places;
assign starting places to each agent;
while $t < 7,200$ **do**
    check if event starts;
    movement of agents;
    contacts (+ new infections);
    ensure transition for health states;
    check for lockdown initiation or end of lockdown;
    if *lockdown* **then**
        update which places are closed;
        move agents to home if necessary;
        ensure that agents wear masks depending on current place;
    end
    if *lockdown ends* **then**
        set all places as open;
        remove masks for agents;
    end
    t = t+1;
end

Algorithm 1: Overview of the simulation process.
